# Assessing primary health care facilities in six prioritized Nigerian states: Linking facility readiness with service availability

**DOI:** 10.1371/journal.pone.0360425

**Published:** 2026-10-13

**Authors:** Olufunke Fasawe, Abubakar Abba, Abubakar Yerima Mohammed, Cynthia Kukoyi, Kabiru A. Gulma, Ashiru A. Abubakar, Abdulrahman Shuaibu, Bello Jamoh, Mohammed Nasir Mahmoud, Yahaya Shamsudeen, Ibrahim A. Mustafa, Ibrahim A. Dangana, Abubakar Yakubu, Ahmed Sani, Babawale Peter, Oyewole Oladapo, Nazeef Mohammed, Nasir Umar, Lawrence N. Anyanwu, Adebowale S. Ayo

**Affiliations:** 1 Clinton Health Access Initiative (CHAI), Abuja, Nigeria; 2 Sector-Wide Approach Coordinating Office, Federal Ministry of Health and Social Welfare, Abuja, Nigeria; 3 Gombe State Primary Health Care Development Agency, Gombe, Nigeria; 4 Kaduna State Primary Health Care Board, Kaduna, Nigeria; 5 Ahmadu Bello University Zaria, Zaria, Nigeria; 6 Kano State Primary Health Care Management Board, Kano, Nigeria; 7 Katsina State Primary Health Care Development Agency, Katsina, Nigeria; 8 Lagos State Primary Health Care Board, Lagos, Nigeria; 9 Niger State Ministry of Primary Health Care, Minna, Niger State, Nigeria; 10 Department of Epidemiology and Medical Statistics, Faculty of Public Health, College of Medicine, University of Ibadan, Ibadan, Nigeria; 11 Population Training and Health Research Entity, North-West University, Mafikeng, South Africa; LMU München: Ludwig-Maximilians-Universitat Munchen, NEPAL

## Abstract

**Background:**

Nigeria’s poor health indicators have been linked to its dysfunctional Primary Health Care (PHC) facilities. Policy gaps remain in achieving sustainable and equitable provision of PHC for Nigerians. Therefore, this study examined the relationship between health facilities’ readiness and service provision in Nigeria.

**Methods:**

This was a cross-sectional assessment of 204 PHC facilities enrolled under the Basic Health Care Provision Fund program, in six prioritized states in Nigeria. The facilities were assessed for the provision of ≥80% of the three levels of primary healthcare services—Tier 1, Tier 2, Tier 3—as the outcome variables. The three tiers represent the minimum, intermediate, and comprehensive levels of essential PHC services, respectively, defined under Nigeria’s Minimum Service Package. Health Facility (HF) readiness (explanatory variable) was assessed across six domains, with five of the domains disaggregated into 3 levels (poor, fair, and good). Data were analysed using Principal Component Analysis (PCA) and multivariable logistic regression model *(alpha* = 0.05).

**Results:**

The majority (81.9%) of the HFs have a functional management committee, 54.4% have an updated BHCPF annual quality improvement plan, 81.9% have standard operating procedures. While 16.2% met the World Health Organization standard ≥2.3 Health Care Workers (HCWs)/1000 population, none met the Minimum Service Package standard for HCWs across all cadres. The proportion of facilities with good readiness in each domain was: management and finance (42.6%); Health Management Information System tools/registers, supply chain tools/registers, and guidelines (40.2%); infrastructure and utility (33.3%); tracer equipment (34.8%); and tracer drugs (29.9%). The percentage of HF that provided ≥80% services was: Tier 1 (65.2%), Tier 2 (46.6%), and Tier 3 (35.8%); while 31.9% met ≥ 80% across all the service Tiers. Common predictors of providing ≥80% across the service Tiers were tracer drugs and equipment availability. The likelihood of delivering all Tiers of services was higher in HF with fair (aOR=2.38, 95% CI = 1.92-11.14, p < 0.05) and good (aOR=3.93, 95% CI = 1.96-8.49) levels of tracer equipment readiness compared to the poor.

**Conclusion:**

Good PHC facility readiness supports the provision of health care services in Nigeria. Availability of tracer drugs and equipment predicts the provision of healthcare services in PHC facilities. Investment in PHC facility functionality and prioritization of funds for tracer drugs and equipment will therefore improve service availability and delivery in Nigerian public PHC facilities.

## Background

Primary Health Care (PHC) aims at ensuring the highest possible level of health and its equitable distribution by focusing on the key population health needs along the continuum from health promotion and disease prevention to treatment, rehabilitation, and palliative care, and as close as feasible to people’s everyday environment [1]. It is rooted in a commitment to social justice, equity, solidarity, and participation, and acknowledges access to the best health care services as a fundamental human right. The delivery of quality, equitable, and dignified PHC services that align with the population’s needs requires availability of a skilled and motivated health workforce, an active supply system, stable infrastructure and utility, and tracer equipment/essential drugs [1]. However, these expectations are yet to be fulfilled in Nigeria, largely attributable to multidimensional factors, including low financial commitment and lack of political will on the part of the government [2]. The federal budgetary allocation to health in Nigeria has witnessed an increment in the past few years, but the level achieved has not met the WHO 15% target for Nigeria’s annual budget for health after the Abuja Declaration, signed by African Union countries in 2001. However, some states in Nigeria have met the threshold [3]. The suboptimal performance of the Nigerian PHC system is of great concern to the government and its international partners [4,5].

Compared with other countries, Nigeria ranks low on nearly all PHC performance indicators [6], which remain far from expectations, especially those that relate to Sustainable Development Goal 3 (SDG 3) [6]. The UHC service coverage index — a weighted average of service coverage, capacity and access — was 91% in Canada, 88% in the UK, 87% in Norway, 86% in the USA and China, 71% in South Africa, 53% in Kenya, 48% in Ghana and 38% in Nigeria [7,8]. The deteriorating health care system in Nigeria has contributed substantially to the country’s low immunization coverage (39%), low modern contraceptive prevalence rate among married (15.3%) and sexually active unmarried women (38%), low life expectancy (55 years), high infant mortality rate (63 deaths per 1,000 live births), and high under-five mortality rate (110 deaths per 1,000 live births), with 40% of under-five children stunted, 8% wasted, and 27% underweight health care [9]. Many Nigerian children do not have access to the necessary nutritional support from the PHC, especially in the provision of vitamin A supplements [9]. These myriad health challenges are avoidable and underscore the need for a strong and sustainably financed PHC system that delivers an integrated package of essential health services.

The Nigerian government took initiatives through health policies and programmes to alleviate its poor health indices. Health reforms were also engaged in sustainable financing to improve PHC performance. These include Primary Health Care Under One Roof (PHCUOR), 2011; Minimum Standards for PHC, 2012; National Health Act, 2014; Basic Health Care Provision Fund (BHCPF), 2018; National Health Insurance Authority (NHIA) Act, 2022; and Presidential Health Reform, 2023 [10]. The presidential health reform, particularly the Nigeria Health Sector Renewal Investment Initiative (NHSRII) through the Sector-Wide Approach (SWAp) — a coordination mechanism that brings all efforts under one agreed plan, one budget, and one monitoring system — to address fragmented resource tracking, service delivery gaps, and weak accountability through the “One Plan, One Budget, One Report, and One Conversation” framework [11]. These initiatives are designed to provide essential PHC services to improve health facilities’ operational capacity and deliver high-quality services. Despite putting these measures in place, many PHC facilities lack the infrastructure and utilities, human resources for health, equipment and drugs, and facility management and finance capacity to adequately and efficiently optimize the benefits of these resources [12]. To address this challenge, Global Alliance for Vaccines and Immunization (GAVI), through Clinton Health Access Initiative (CHAI), is conducting implementation research to test and evaluate the use of Collaborative Learning and pay-for-performance approaches to improve data use for action in selected PHC facilities across 6 prioritized states in Nigeria. This is premised on the hypothesis that the appropriate use of data for action will improve overall PHC performance and, as an outcome, drive equitable coverage of routine immunization and other essential PHC services. This paper emerged from the baseline assessment conducted as part of the larger study being implemented by CHAI. The indicators obtained from this phase shall be used for the programme’s performance evaluation to understand the impact of the project in the short, medium, and long term. It will also facilitate participatory programme development and consultation processes to define the needs, opportunities, and priorities of the health facilities and stakeholders.

Several studies have been conducted on the PHC facilities assessment in Nigeria. Adeosun et al. assessed the drug use in PHC in Lagos State, Nigeria, and found that irrational drug use practices exist in comprehensive and basic PHCs [13]. Oyekale and Juma et al. assessed the PHC facilities’ service readiness in Nigeria and observed a shortage of essential drugs and differences between the availability of basic medical equipment and their functionality [5,14]. Other available studies in Nigeria revealed that most PHC facilities provide sub-optimal services [4,12,15–17]. Existing studies of health service delivery in Nigeria have identified socioeconomic and cultural factors as the primary determinants of health facility delivery [18,19]. However, policy gaps remain in the poor management capacity at the frontline to effectively utilize the available resources and improve facility readiness to provide the needed health care service. The need to examine the relationship between facility readiness and service provision, as provided in the current study, remains a grey area for research in Nigeria. Assessing PHC service delivery from six readiness domains distinguishes the current research from the earlier studies. Therefore, the objectives of the study are to: describe key facility readiness areas (management and finance; HMIS tools/registers, supply chain tools/registers, and guidelines; human resource for health; infrastructure and utility; tracer equipment; and tracer drugs), assess PHC facility readiness for implementing the MSP, examine the association between facility readiness and provision of each of the three tiers of services, and identify the predictors of health services’ provision in the Nigerian PHC facilities. The findings from this study were used to develop a pre-intervention strategy for the main study, identifying key readiness areas to be prioritized for performance improvement and the anticipated improvement in service availability. The outcome of this study and the subsequent interventions provide PHC policymakers with evidence on the impact of improved facility readiness on service availability and health outcomes, which are fundamental ingredients needed to accelerate progress towards UHC and the health-related SDGs in Nigeria.

## Methods

### Study area

This study was conducted in six prioritized states in Nigeria: Gombe, Kaduna, Kano, Katsina, Lagos and Niger. According to the 2006 Population and Housing Census, the country’s population was 140,431,790. Using a projected annual growth rate of 2.47%, Nigeria’s population was estimated at 201,135,262 in 2020. Nigeria is made up of 36 states and a Federal Capital Territory (FCT), grouped into six geopolitical zones: North Central, North East, North West, South East, South-South, and South West. The 2020 estimated population figure for each state was Gombe (3,623,462), Kaduna (8,324,285), Kano (14,253,549), Katsina (9,300,382), Lagos (12,772,884), and Niger (6,220,617) [20].

Facility selection was guided by three criteria: zero-dose vaccination burden, GAVI priority state designation, and active BHCPF implementation [21]. The six states included in the study were selected from among those with the highest zero-dose burden in Nigeria — defined as the number of children aged 12–23 months who had not received the first dose of pentavalent vaccine (Penta 1), a proxy for unimmunized and underserved populations [22]. Selected states were also required to have existing GAVI investments to complement project interventions and maximize sustainability, and to be actively implementing the BHCPF program, ensuring facilities had access to predictable financing to address identified readiness gaps.

The health care system in each of these six states is based on the three-tier system of primary, secondary, and tertiary care. The State’s Ministry of Health and other health partners, including the primary health care development agency, are responsible for the development of policies, strategic plans, guidelines, and programmes that provide the overall direction for the health care service delivery system. Through resource mobilization, collaboration, partnership, and development of community-based financing projects and functional health facilities, the State Ministry of Health (SMoH) ensures the delivery of free quality primary health care services to its citizens within the state. This includes promoting, preventing, rehabilitating, and providing curative health care services, in line with the National Health Policy and the Nigerian Constitution. They also formulate, monitor, and evaluate health policies and strategies, and supervise health services within the state.

### Study population

The study involved 204 PHC facilities implementing the BHCPF program. Each facility was assessed through interviews with facility managers. However, some segments of the facility assessment questionnaire were administered to the head of the unit where the information was required. An observational approach was used to verify the authenticity of the information provided, especially the availability of tools, physical structure, equipment, and drugs.

### Study design

This facility assessment study adopted a cross-sectional design approach. An interviewer-administered questionnaire was used to collect data on general facility information, infrastructures and utilities, administrative section, service delivery, laboratory services, human resources mapping, instruments and equipment, supply chain management and commodities availability, maternal and newborn service delivery, child health service delivery, family planning service delivery, clinical service utilization, and financial management (Fig 1).

**Fig 1:**
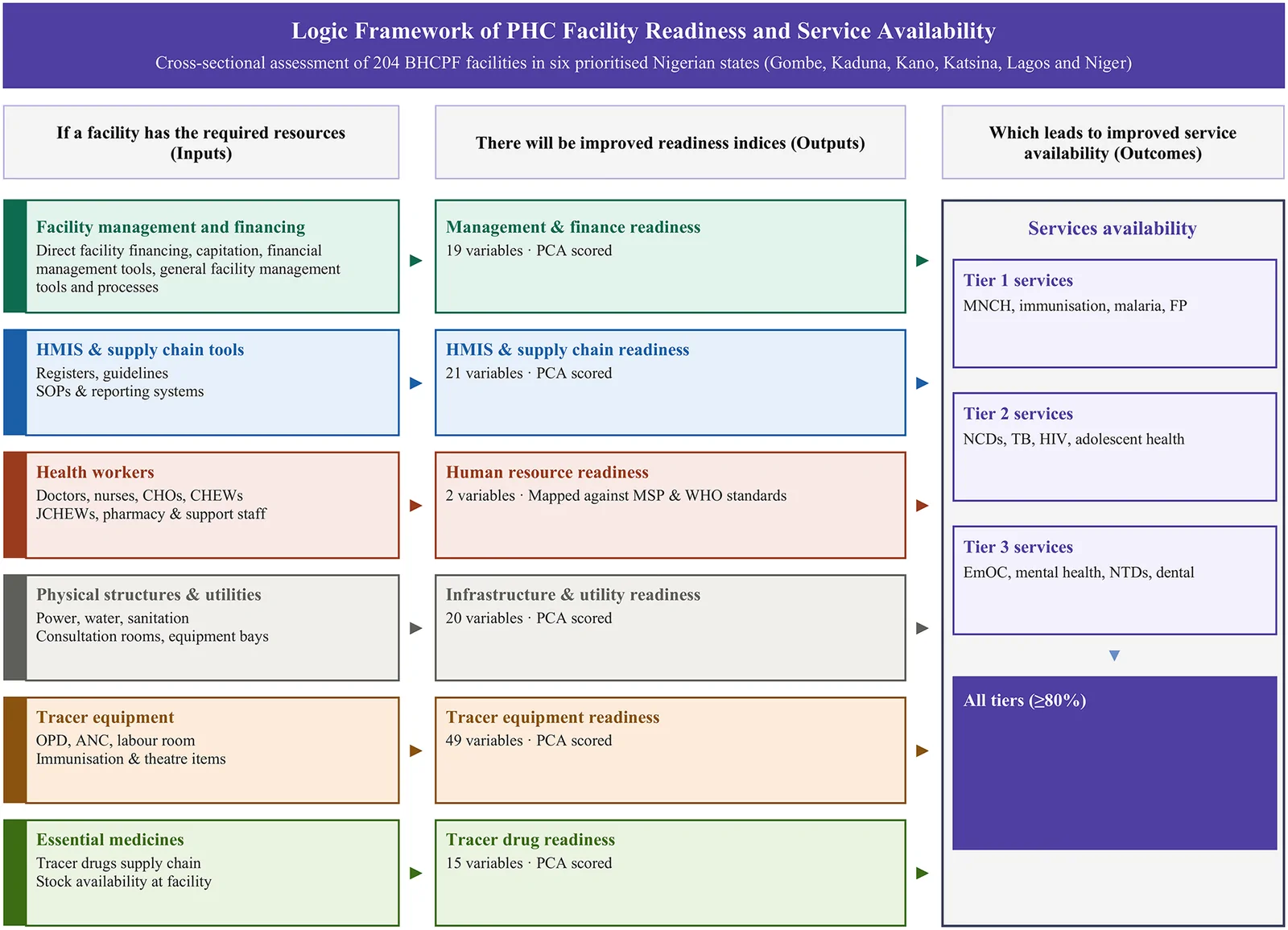
Logic model of PHC facility readiness performance and service availability. Fig 1 describes the framework that illustrates the hypothesized relationship between facility-level resource inputs, readiness indices (outputs), and health service availability (outcomes) across 204 BHCPF-enrolled facilities in six prioritized Nigerian states. Six input domains — facility management and financing, HMIS and supply chain tools, human resources for health, infrastructure and utilities, tracer equipment, and essential medicines — are each linked to a corresponding readiness index derived through Principal Component Analysis (PCA). These indices, in turn, are posited to predict the availability of Tier 1 (primary), Tier 2 (intermediate), and Tier 3 (advanced) services, as well as performance across all tiers combined.

### Sampling procedure

A list of 1720 BHCPF facilities was mapped across the 6 states. Eighty-eight (88) qualified facilities were removed from the list due to security challenges at their location at the time of the study, thus reducing the number of facilities to 1632 PHCs. A sample of 204 PHC facilities was selected based on the estimated sample size for the study.

### Training of the data collectors

Fifty-two (52) data collectors with a minimum of a bachelor’s degree in medicine, nursing and related fields, who have fieldwork experience and knowledge of the local language received training at the state level. The focus of the training was on the discussion of concepts, the process of data collection, and methodologies for conducting a survey. In addition, break-out sessions, in-class exercises, case studies, and presentations were held daily, at which trainees had the opportunity for hands-on practice and role play. The supervisors received additional training on how to provide logistic support, manage field teams, observe interviews, and keep an inventory of supplies. Field editors received additional training on supervisory activities, including operation of the Computer-Assisted Personal Interviews (CAPI) system. Field practice was conducted at each level of the training. A pre-test was conducted at a location similar in characteristics to the study areas, but outside the boundary of the study areas, to ascertain the internal consistency, reliability, and validity of the questionnaire before use for the main data collection exercise. CAPI, programmed on SurveyCTO, was used for data collection, and it was coded to accept only valid responses, automatically perform checks, skip to the proper question based on the provided responses, and perform a consistency check for the data collected. The data collectors received training on how to protect themselves and their respondents from any negative reactions from community members, where such exist.

### Data collection

A semi-structured interviewer-administered questionnaire and checklist were used for data collection. PHC facilities performance assessment tools were adapted from the tools previously used for assessing the performance of primary care in Nigeria [4]. The questions in the questionnaire were generated after an exclusive literature search and review of the relevant available documents. The development of the questionnaires also benefited from the opinions and inputs from local experts and academics in the field. Data collection was carried out from 5^th^–14^th^ February, 2024. Data collectors conducted advocacy visits to community gatekeepers and Local Government Level primary healthcare directors to discuss the aim and ethics of the assessment and secure their buy-in before the commencement of the fieldwork. This was in addition to approvals obtained from the respective State Primary Healthcare Boards/Agencies, and the advocacy visits were carried out at all study sites across the six states. The data collectors complied with all the ethical rules in the data-gathering process. The administration of the questionnaire took up to 2 hours. Whenever the security of data collectors was threatened, they were advised to discontinue the interviews at those sites until normalcy returned to the area.

### Variables description

The outcome variable was service delivery, segregated into three groups, namely, Tier 1, Tier 2, and Tier 3 services. Facilities were assessed based on whether they provided at least 80% of services in each tier (Tier 1, Tier 2, and Tier 3) and at least 80% of all services combined. A detailed description of the tiers of PHC services in Nigeria is outlined in S1 Table.

The main independent variable was facility readiness. Facility Readiness Index (FRI) was assessed based on questions on six (6) primary domains (Table 2). Each question was based on the item’s availability, with a response coded as 1 if the item is available, and 0 if otherwise. An index of readiness was computed for five of the six domains using the Principal Component Analysis (PCA) technique. These are: health facility management and financing; HMIS tools/registers, supply chain tools/registers, and guidelines; infrastructure and utility; availability of tracer equipment; and availability of tracer drugs. PCA was chosen among other data reduction methods because it simplifies complex data while maintaining its key features. We ensured that the primary assumptions of PCA, such as linearity, normality, independence, and correlation, were not violated. The PCA-generated index score (quantitative) [20] was disaggregated into three groups, using the 2^nd^ and 3^rd^ percentiles of the overall score as thresholds, i.e., scores that are less than 50%, at least 50% but less than 75%, and at least 75% were categorized as poor, fair, and good, respectively [23,24]. Percentile-based thresholds provide a distribution-based approach for defining relative categories within a study population. A percentile cut-off was selected because it provides a distribution-based criterion that does not require assuming a particular underlying distribution [23,24]. However, for the human resources for health, the score for each facility was generated in quantitative terms. Each facility was also assessed to determine whether it met the number of staff required in each cadre of health care worker, as outlined in S2 Table.

### Data management and analysis

Data entry and editing were carried out using the CSPro software package. Data analyses, including recoding, variable generation, cross-tabulations, and statistical tests, were conducted using Stata software version 15.0 and Excel. The Chi-square test was used to examine the association between each facility readiness domain and the provision of health services. A multivariable logistic regression model was used to identify the predictors of at least Tier 1, Tier 2, Tier 3, and all Tiers of services by the PHC facilities. Unlike other regression models, logistic regression does not require linearity, normality, and homoscedasticity assumptions. Multicollinearity was assessed among the six readiness domains, and no multicollinearity issues were observed among the explanatory variables. The dependent variables were dichotomous, and observations were assumed to be independent. All tests were conducted at a 5.0% level of significance.

### Ethical consideration

Ethical clearance was sought and obtained from the National Health Research Ethics Committee of Nigeria (NHREC) and State Ethics Committees (NHREC approval number: NHREC/01/01/2007–15/01/2024). To access organizations during the data collection, permissions were obtained from the appropriate gatekeepers. Written informed consent was obtained from the respondents, and the anonymity and confidentiality of the information they provided were assured. At the start of each interview, participants were provided with a consent statement explaining the study’s purpose. They were informed of their right to withdraw at any stage without consequence. Each participant read and signed the consent statement before commencing the interview. Data collection devices and relevant paper documents were protected from misuse or theft by storing them in locked areas. Backups were created for the data. For confidentiality, data files were encrypted to make them unreadable to anyone except the authorized individuals who have the appropriate password and multi-factor authentication. The data access list is reviewed periodically to promptly revoke access, when necessary. The permission to use the data for publications or other engagements is official and can only be granted by the implementing partners.

## Results

### Health facility management and financing

The data, as presented in Table 1, showed that almost all the facilities (98.0%) have a functional Ward Development Committee (WDC), while 167 (81.9%) have a functional health facility management committee, 122 (59.8%) have an updated staff list with name and cadre, and 110 (53.9%) have an organogram showing responsible persons and hierarchy structure. About 54.4% and 49.5% of the health facilities have an updated BHCPF annual quality improvement plan and updated expenditures aligned with the business plan, respectively. 162 (79.4%) have received monthly BHCPF capitation from State Health Insurance Authorities (SHIA).

**Table 1:** Percentage distribution of health facilities (n = 204) according to health facility management and financing readiness components.

Availability	Freq.	%	95% (CI)
**Organization and Management**			
Functional Ward Development Committee (WDC)	200	98.0	96.1–99.9
Functional HF Management Committee (MC)	167	81.9	76.5–87.2
Receipt of Monthly BHCPF capitation from SHIA	162	79.4	73.8–85.0
Receipt of Quarterly BHCPF capitation from State Primary Health care	152	74.5	68.5–80.5
**SPHCB**			
The BHCPF Facility quality scorecard is visibly displayed	136	66.7	60.1–73.2
Setting up of BHCPF Quality Improvement Team	19	9.3	5.2–13.3
**Documentation and Organizational Resources**			
Updated staff list with name and cadre	122	59.8	53.0–66.9
Organogram showing responsible persons and hierarchy structure	110	53.9	47.0–60.8
Staff distribution list (with cadre) clearly pasted within the facility	105	51.5	44.5–58.4
Updated check-in and check-out attendance register/biometry	123	60.3	53.5–67.1
Updated and visibly displayed Duty roster	133	65.2	58.6–71.8
**Budgeting & Expenditure Tracking**			
Updated BHCPF Annual Quality Improvement Plan	111	54.4	47.5–61.3
Updated Costed BHCPF Quarterly Business Plan	111	54.4	47.5–61.3
Updated Expenditures Aligned with the Business Plan	101	49.5	42.5–56.4
**Financial Documentation**			
Updated petty cash book	102	50.0	43.0–56.9
**Accounting System Practices**			
Developed a monthly financial report (MFR) for a service account	138	67.6	61.1–74.1
Developed MFR for SCHMA/SHIA funds (excluding BHCPF)	97	47.5	40.6–54.5
Developed quarterly financial reports BHCPF	139	68.1	61.6–74.6

### Health management information system (HMIS) tools/registers, supply chain tools/registers, and guidelines

The data showed that above 98% of the health facilities have a monthly summary form, a daily general attendance register, and an outpatient department (OPD) register. The availability of ANC register, standard operating procedures (SOPS), protocols or guidelines, and child immunization register was found in 194 (95.1%), 167 (81.9%), and 199 (97.5%) health facilities, respectively. The availability of guidelines such as Basic Emergency Obstetric and Newborn Care (BEmONC)/ Comprehensive Emergency Obstetric and Newborn Care (CEmONC), neonatal resuscitation, and integrated management of childhood illnesses 108 (52.9%), 126 (61.8%), and 159 (77.9%), respectively; while 188 (92.2%) have an immunization schedule guideline, 180 (88.2%) have a family planning guideline (Table 2).

**Table 2:** Frequency and percentage distribution of facilities according to the availability of HMIS tools/registers, Supply Chain tools/registers, and Guidelines.

Availability	Freq.	%	95% (CI)
**HMIS tools/registers**			
Monthly summary form	201	98.5	96.8–100.2
Daily general attendance register	201	98.5	96.8–100.2
ANC register	194	95.1	92.1–98.1
Labour and delivery register	148	72.5	66.3–78.7
Map/list of SHF/Tertiary facilities to which patients are referred	112	54.9	48.0–61.8
Use of triplicate referral forms	150	73.5	67.4–79.6
Child immunization register	199	97.5	95.4–99.7
**Supply Chain Management Tools**			
Tally sheets	130	63.7	57.0–70.4
Inventory control card (ICC)/Stock cards	146	71.6	65.3–77.8
Bin Card	139	68.1	61.6–74.6
Combined Report and Requisition Form (CRRF)	123	60.3	53.5–67.1
Daily Consumption Register (DCR)	145	71.1	64.8–77.4
Staff responsible for managing the ordering of EDMC	171	83.8	78.7–88.9
OPD register	200	98.0	96.1–99.9
Postnatal care register	147	72.1	65.8–78.3
**Guidelines**			
BEmONC/CEmONC guidelines	108	52.9	46.0–59.9
Standard Operating Procedures (SOPs), protocols, or guidelines	167	81.9	76.5–87.2
Neonatal resuscitation guidelines	126	61.8	55.0–68.5
Integrated Management of Childhood Illnesses guidelines	159	77.9	72.2–83.7
Immunization schedule guidelines	188	92.2	88.4–95.9
Family planning guidelines	180	88.2	83.7–92.7

### Human resources for health

Across the 204 facilities examined, only 33 (16.2%) met the minimum skilled HCWs density (WHO standard (2.3 HCWs/1000 population)) to achieve 80% skilled birth attendant coverage. The distribution of cadres of health care workers according to the national MSP standard for health care workers is displayed in Fig 2. The data showed that 35 (17.2%) of the health facilities have at least 1 Medical Doctor, 78 (38.2%) have at least 1 CHO, 75 (36.8%) have at least 4 Nurses/Midwives, 149 (73.0%) have at least 3 CHEWs, and 25 (12.3%) have at least 6 JCHEWs. The frequency of facilities with at least 1 Environmental Health Officers (EHO), at least 2 Pharmacy Technicians, at least 2 Laboratory Technicians, and at least 2 Health Attendants (HA) was 105 (51.5%), 44 (21.6%), 91 (44.6%), and 77 (37.8%), respectively; while 26 (12.7%) have at least 2 Medical Record Officers (MRO) and 95 (46.6%) have at least 2 security staff (Fig 3).

**Fig 2:**
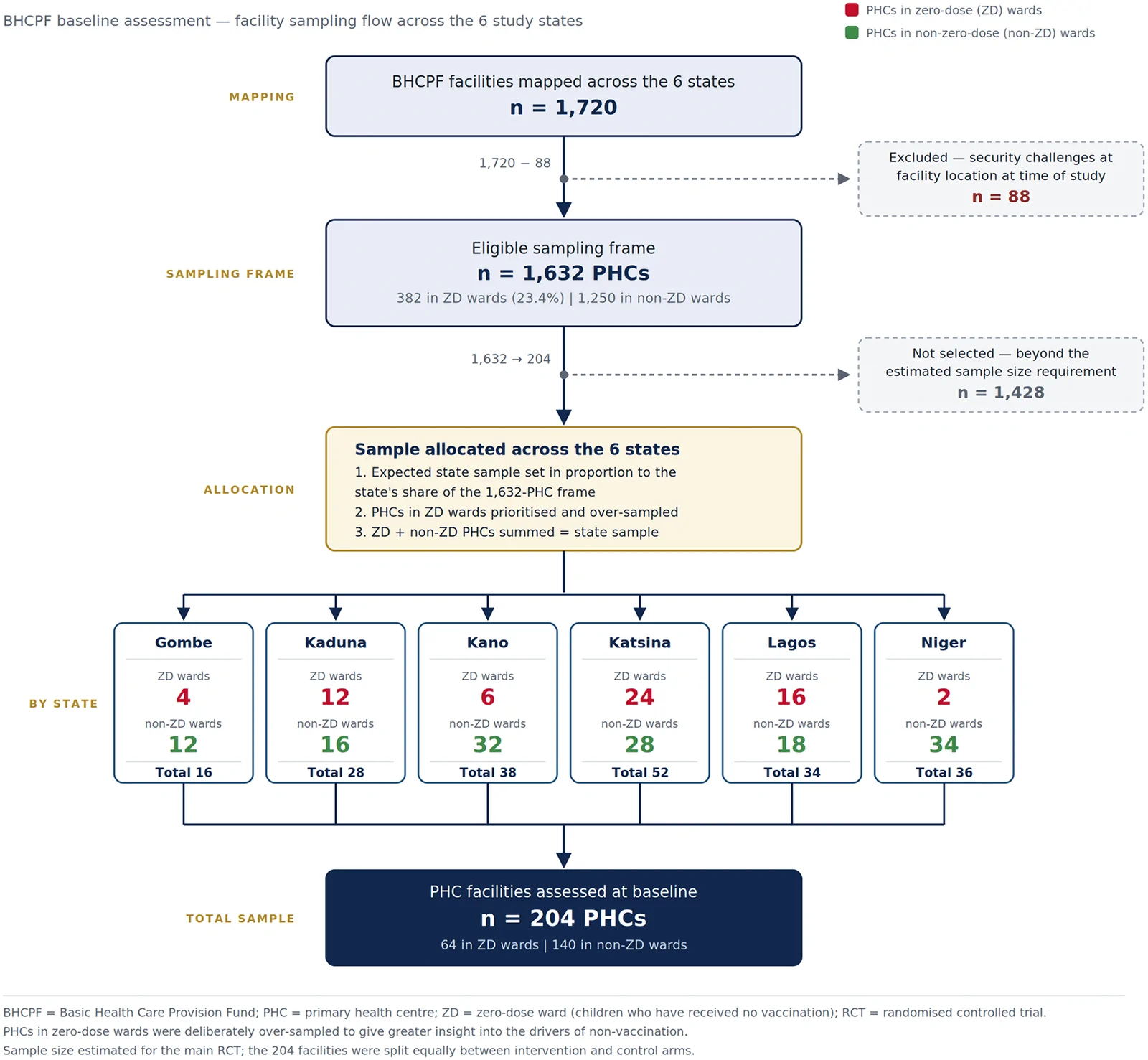
Process flow of the facility sample selection procedure. The proportion of facilities with no zero-dose was also calculated per state. However, the proportion of zero-dose facilities in the sample was prioritized and doubled to gain more insights into the problems associated with no vaccination in these facilities. Thereafter, the calculated number of zero-dose facilities and the number of facilities with no zero-dose per state were added to produce the total number of facilities sampled in that state. Thus producing 16 facilities in Gombe, 28 in Kaduna, 38 in Kano, 52 in Katsina, 34 in Lagos, and 36 in Niger. The examined facilities were randomly selected from the list of PHCs allotted to each state.

**Fig 3:**
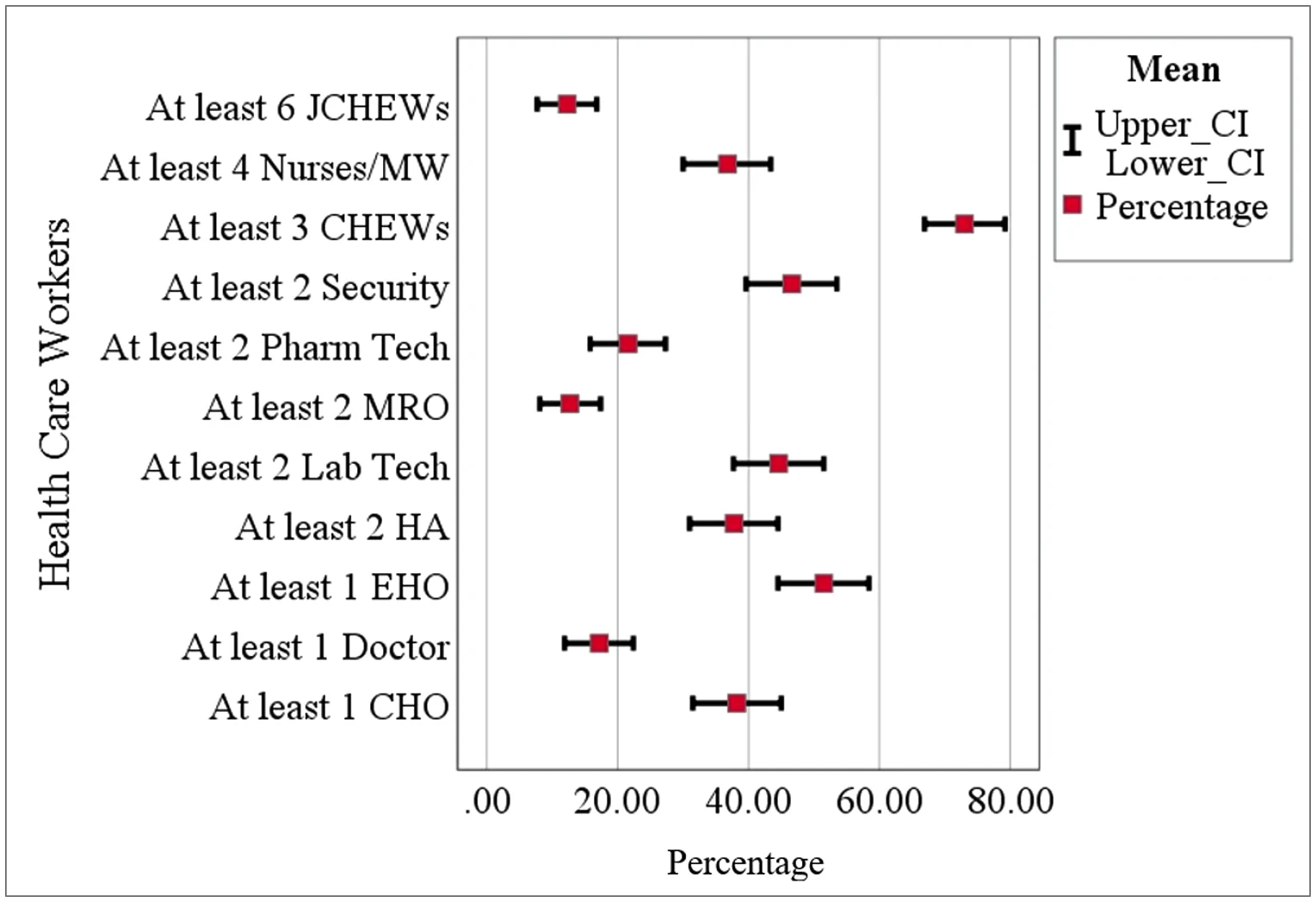
Distribution of the availability of cadres of health care workers according to the MSP standard for health care worker.

Thirteen health facilities (6.4%) did not meet the MSP standard for any cadre of HCWs, while 20 (9.8%), 32 (15.7%), and 38 (18.6%) met the standard for only one, two, and three cadres, respectively. About 7.8% of the health facilities met the MSP standard for five cadres, whereas the MSP standard for HCWs was met for seven cadres in 13 (6.4%) of the health facilities. None of the health facilities met the MSP standard for HCWs in all eleven cadres of HCWs (Fig 4).

**Fig 4:**
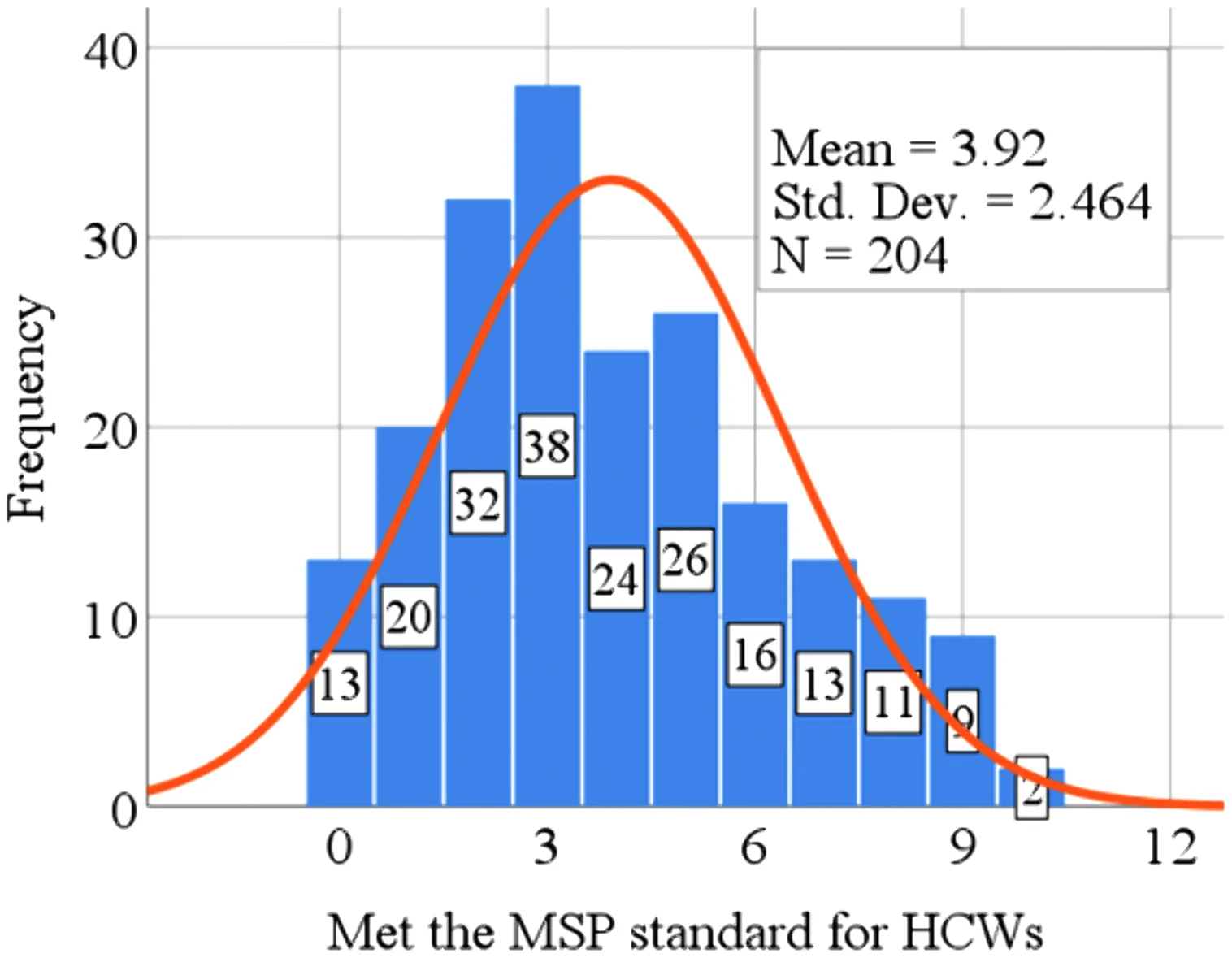
Frequency distribution of health facilities that met the MSP standard for health Care Workers.

In Fig 5, the data showed that training on financial or operational management was received by at least two staff in 94 (46.1%) facilities, while at least 2 staff have received training on accounting, budgeting, and retirement in 108 (52.9%), 116 (56.9%), and 117 (57.4%), respectively.

**Fig 5:**
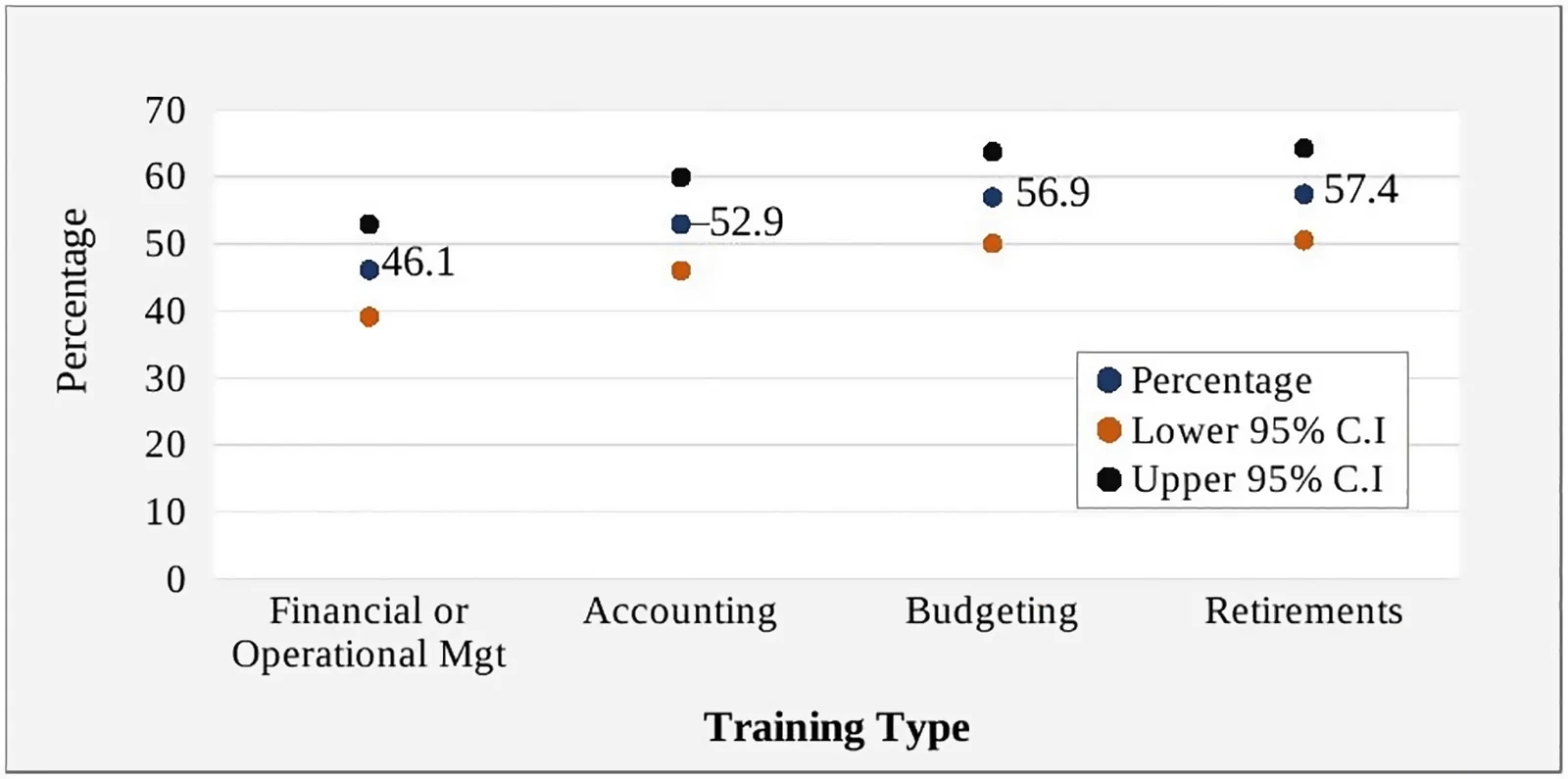
Distribution of health facilities according to the training received by at least two staff on financial/operational management, budgeting, and the retirement of documents.

### Infrastructure and utility

The data, as shown in Table 3, depict that 48 (23.5%) of the health facilities have accommodation in/around the facility or elsewhere for workers, 141 (69.1%) have well-netted and burglary-protected windows, and 153 (75.0%) have clearly labelled service points. About 147 (72.1%) facilities have handwashing facilities for staff with soap & running water, 131 (64.2%) have handwashing facilities for patients and visitors with soap & running water, 168 (82.4%) have functioning toilet facilities or latrine, and 136 (66.7%) have separate toilets for both clients and staff. Functioning generator set for backup electricity was found in 113 (55.4%) of health facilities and 46.6% of health facilities have functioning solar power, 111 (54.4%) have an emergency light or lamp, 160 (78.4%) have a secure storage area for commodities and consumables, 24 (11.8%) have a functional and dedicated ambulance, and 123 (60.3%) have functional means of communication with clients/stakeholders.

**Table 3:** Distribution of the health facilities (n = 204) according to the availability of infrastructure and utility required for functional PHC.

Availability	Freq.	%	95% CI
Accommodation in/around or elsewhere the facility for workers	48	23.5	17.6–29.4
Well-netted and burglary-protected windows	141	69.1	62.7–75.5
Clearly labelled service points	153	75.0	69.0–80.9
Water source	133	65.2	58.6–71.7
Well-covered water storage tank/overhead tank	116	56.9	50.0–63.7
Handwashing facilities (HF) for staff with soap & running water	147	72.1	65.8–78.3
HF for patients and visitors with soap & running water	131	64.2	57.5–70.9
Functioning toilet facilities or latrine	168	82.4	77.0–87.6
Separate toilet for both clients and staff	136	66.7	60.1–73.2
Functioning generator set for backup electricity	113	55.4	48.5–62.3
Functioning solar power	95	46.6	39.6–53.5
Emergency light or lamp	111	54.4	47.5–61.3
Secure storage area for commodities and consumables	160	78.4	72.7–84.1
Refuse disposal bin at all service points	178	87.3	82.6–91.9
Refuse disposal bins with a lid in all wards	134	65.7	59.1–72.3
Functional and dedicated ambulance	24	11.8	7.3–16.2
Access to any vehicle for emergency transport services	72	35.3	28.6–41.9
Functional means of communication with clients/ stakeholders	123	60.3	53.5–67.1
Functional computer	55	27.0	20.8–33.1
Protected Filing System/Cabinet	154	75.5	69.5–81.4

### Availability of equipment and drugs

In Table 4, the data depict that the majority of the health facilities have an examination couch (68.6%), a sphygmomanometer (76.5%), a stethoscope (81.9%), a clinical thermometer (71.1%), a weighing scale (76.0%), and a Shakir’s strip (71.1%) in their OPD unit, while 48.5% and 14.7% have a screen and a pulse oximeter in the unit. The most common equipment found in the immunization unit of the health facilities was a functional safety box (98.5%), followed by a vaccine carrier (94.6%), and a Shakir’s strip (78.4%). In the ANC unit, the number of facilities with at least one functional equipment was 153 (75.0%) weighing scales, 136 (66.7%) sphygmomanometers, 143 (70.1%) stethoscopes, 180 (88.2%) tape measures, 161 (78.9%) pinnard stethoscopes/fetoscopes, and 167 (81.9%) examination couches.

**Table 4:** Distribution of health facilities according to availability of at least one functional tracer equipment in the specific unit of the health facilities.

Unit/Equipment	Freq.	%	95% (CI)	Unit/Equipment	Freq.	%	95% (CI)
**OPD**				Needle holding forceps	114	55.9	49.0-62.8
Examination Couch	140	68.6	62.2–75.1	Dissecting Forceps	100	49.0	42.1–55.4
Screen	99	48.5	41.6–55.5	Artery Forceps	113	55.4	48.5–62.3
Sphygmomanometer	156	76.5	70.6–82.3	Drip Stands	103	50.5	43.5–57.4
Stethoscope	167	81.9	76.5–87.2	Sterilizers	52	25.5	19.4–31.5
Clinical Thermometer	145	71.1	64.8–77.4	Angle Poise Lamp	25	12.3	7.7–16.8
Weighing Scale	155	76.0	70.0–81.9	Ambu Bag and Mask	39	19.1	13.6–24.6
Pulse Oximeter	30	14.7	9.8–19.6	Nebulizer	19	9.3	5.2–13.3
Shakir’s Strip	145	71.1	64.8–77.4	**Labor Room**			
**Immunization Unit**				Delivery Bed	144	70.6	64.2–76.9
Weighing Scale	141	69.1	62.7–75.5	Delivery Tray	126	61.8	55.0–68.5
Shakir’s Strip	160	78.4	72.7–84.1	Artery Forceps	136	66.7	60.1–73.2
Vaccine Carrier	193	94.6	91.4–97.7	Dissecting Forceps	120	58.8	52.0–65.6
Safety Box	201	98.5	96.8–100.0	Epsiotomy Scissors	108	52.9	46.0–59.9
Gali Pot	115	56.4	49.5–63.2	Gali Pot	129	63.2	56.5–69.9
Kidney Dish	106	52.0	45.0–58.9	Kidney Dish	143	70.1	63.7–76.4
**ANC**				Speculum	118	57.8	51.0–64.7
Weighing Scale	153	75.0	69.0–81.0	Pinnard Steth./Fetoscope	128	62.7	56.0–69.4
Sphygmomanometer	136	66.7	60.1–73.2	Drip Stands	141	69.1	62.7–75.5
Stethoscope	143	70.1	63.7–76.4	Mckintosh	94	46.1	39.1–52.9
Pinnard Stethoscope/Fetoscope	161	78.9	73.2–84.6	Non–Pneumatic Anti–Shock Garment	110	53.9	47.0–60.8
Tape Measure	180	88.2	83.7–92.7	MVA Kit	57	27.9	21.7–34.2
Examination Couch	167	81.9	76.5–87.2	Sphygmomanometer	91	44.6	37.7–51.5
Screen	103	50.5	43.5–57.4	Stethoscope	110	53.9	47.0–60.8
Thermometer	97	47.5	40.6–54.5	Urinal	92	45.1	38.2–51.9
**Minor Theatre/Casualty**				Suction Bulb	99	48.5	41.6–55.5
Scissors	134	65.7	59.1–72.3	Ambu Bag and Mask (Neonatal and Adult)	103	50.5	43.5–57.4

### Availability of tracer drugs

The data, as shown in Table 5, revealed that 97 (47.5%) of the health facilities have magnesium sulphate injection, while 131 (64.2%) have misoprostol tabs, and 129 (63.2%) have oxytocin injection. Above 80% of the health facilities have pentavalent (92.2%), folic acid tabs (91.2%), paracetamol syrup (87.3%), tetanus diphtheria vaccine (85.3%), BCG (84.8%), artemisinin combination therapy tabs (84.4%), injectable contraceptives (83.3%), and combined oral pills (83.3%). The most available tracer drug in the health facilities was the pentavalent vaccine (92.2%), and the least was chlorhexidine gel (43.6%).

**Table 5:** Percentage distribution of facilities by availability of tracer drugs.

List of Tracer Drugs	Freq.	%	95% (CI)
Magnesium Sulphate Injection	97	47.5	40.6–54.5
Misoprostol Tabs.	131	64.2	57.5–70.9
Oxytocin Injection	129	63.2	56.5–69.9
Folic Acid Tabs.	186	91.2	87.2–95.1
Sulphadoxine + Pyrimethamine Tabs.	161	78.9	73.2–84.6
Amoxicillin Dispersible/ Susp.	158	77.5	71.6–83.2
Zinc/ORS powder	60	29.4	23.1–35.7
Artemisinin Combination Therapy Tabs.	173	84.8	79.8–89.8
Paracetamol Syrup	178	87.3	82.6–91.9
Chlorhexidine Gel	89	43.6	36.7–50.5
BCG	173	84.8	79.8–89.8
Pentavalent	188	92.2	88.4–95.9
Tetanus Diphtheria Vaccine	174	85.3	80.3–90.2
Combine Oral Pills	170	83.3	78.1–88.5
Injectable Contraceptives	172	84.3	79.2–89.4

In the management and finance domain, 87 (42.6%), 24 (11.8%), and 93 (45.6%) of the health facilities have good, fair, and poor levels of readiness, respectively (Fig 6a), while in the HMIS tools/registers, supply chain tools/registers, and guidelines domain, 82 (40.2%), 58 (28.4%), and 64 (31.4%) of the health facilities have good, fair, and poor levels of readiness concerning availability of HMIS tools/registers, supply chain tools/registers, and guidelines, respectively (Fig 6b). In the infrastructure and utility domain, 68 (33.3%), 62 (30.4%), and 74 (36.3%) health facilities have good, fair, and poor levels of readiness, respectively (Fig 6c). In the domain of tracer equipment, the number of health facilities that were categorized as good, fair, and poor in terms of readiness was 71 (34.8%), 70 (34.3%), and 63 (30.9%), respectively (Fig 6d).

**Fig 6:**
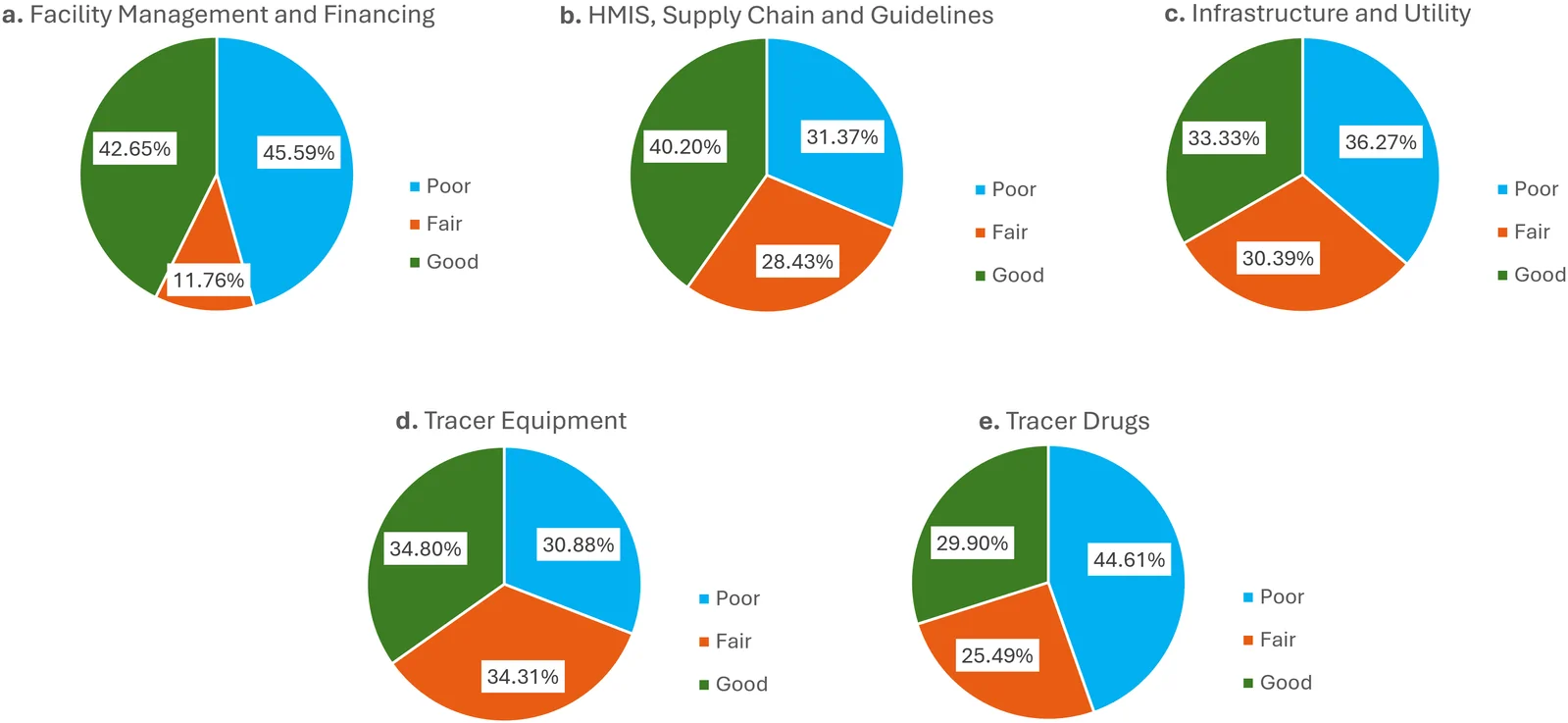
(a-e). Percentage distribution of health facilities according to their levels in each readiness domain. a. Percentage distribution of facilities according to their level of management and finance readiness. b. Percentage distribution of facilities according to their level of HMIS tools/registers, supply chain tools/registers, and guidelines readiness. c. Percentage distribution of health facilities according to their levels of infrastructure and utility readiness. d. Percentage distribution of health facilities according to their levels of at least one functional tracer equipment readiness. e. Percentage distribution of health facilities according to their levels of tracer drug readiness.

### Service delivery

The data showed that 133 (65.2%), 95 (46.6%), and 73 (35.8%) provided at least 80% of Tier 1, Tier 2, and Tier 3 services, respectively. About 32.4% of the facilities did not meet the threshold in any of the service Tiers, while 40 (19.6%) met the service provision threshold in one of the Tiers, and 33 (16.2%) provided 80% services in exactly two of the Tiers. Sixty-five (31.9%) of the facilities met the service threshold across the three Tiers, i.e., they met ≥ 80% in each of the three service Tiers (Fig 7).

**Fig 7:**
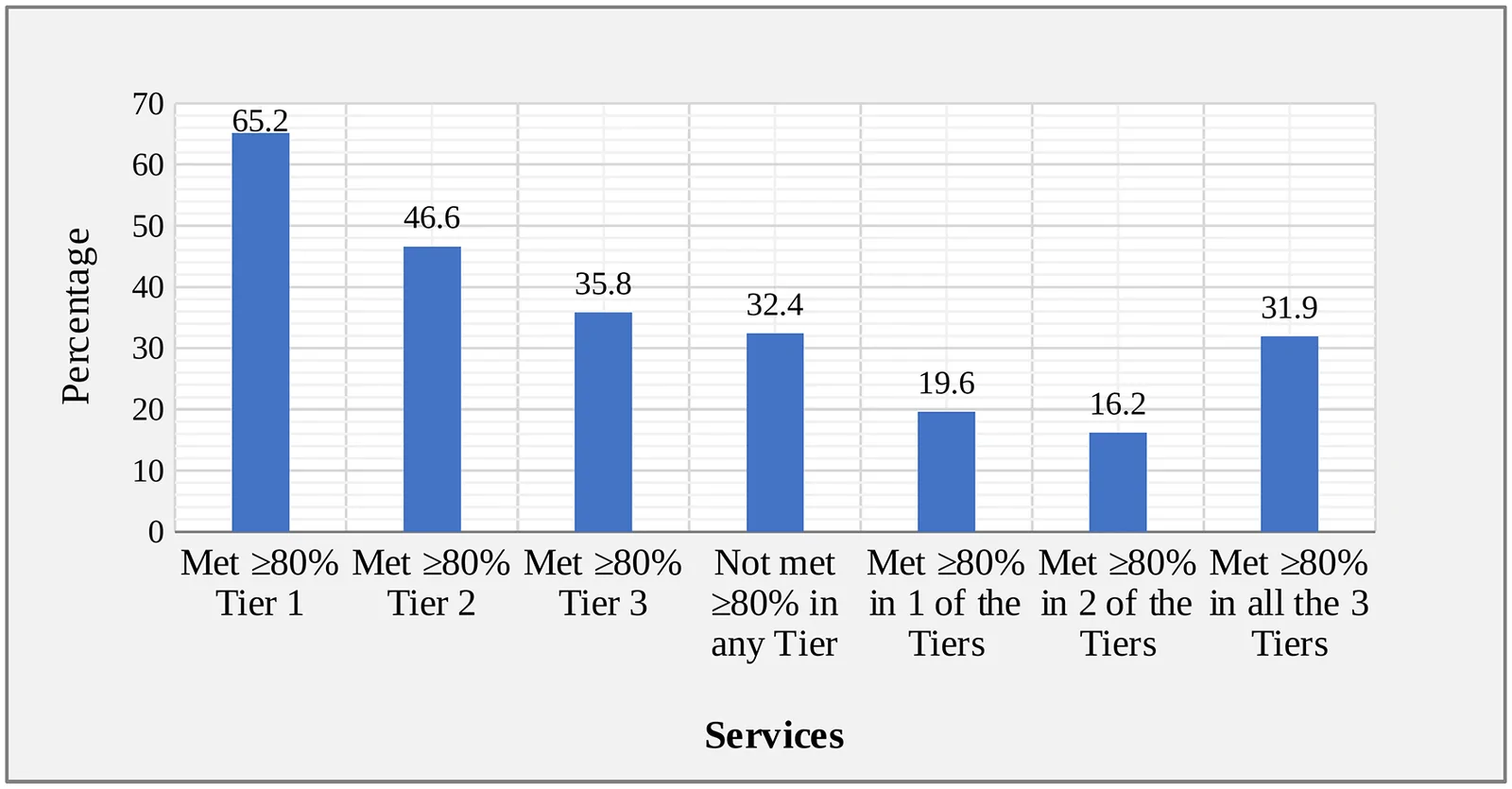
Percentage distribution of health facilities according to proportion Tiers 1, 2, and 3 services provided.

In Table 6, the data showed that variables found to be significantly associated with the provision of at least 80% Tier 1 services include: HMIS, supply chain, and guidelines; met 50% MSP; infrastructure and utility; tracer drugs; and tracer equipment (p<0.05). The provision of at least 80% Tier 1 services increases consistently with the increasing level of availability of HMIS tools/register, supply chain tools/register, and guidelines; infrastructure and utility; tracer drugs; and tracer equipment. The percentage of health facilities that met the service provision level for Tier 1 services was higher among those that met the WHO standard of ≥2.3 health care workers per 1,000 population (78.8%) than those that did not meet this criterion (62.6%) (p>0.05). The facilities that met at least 50% of MSP for health care workers have a higher percentage (90.2%) of providing Tier 1 services than those that did not (56.9%).

**Table 6:** Association between facilities background characteristics and service provision.

Background Characteristic	≥80% in Tier 1 %(n)	(*χ*2-value) Freq.	≥80% in Tier 2 %(n)	(*χ*2-value) Freq.	≥80% in Tier 3 %(n)	(*χ*2-value) Freq.	≥80% in all Tiers %(n)	(*χ*2-value) Freq.
**Total**	65.2(133)	204	46.6(95)	204	35.8(73)	204	31.9(65)	204
**Gender of Officer in Charge**		(3.23)		(0.89)		(4.11)*		(4.15)*
Female	71.4(70)	98	50.0(49)	98	42.9(42)	98	38.8(38)	98
Male	59.4(63)	106	43.4(46)	106	29.2(31)	106	25.5(27)	106
**Facility Management and Financing**		(5.75)		(5.72)		(1.19)		(2.53)
Poor	57.0(53)	93	39.8(37)	93	34.4(32)	93	29.0(27)	93
Fair	79.2(19)	24	66.7(16)	24	45.8(11)	24	45.8(11)	24
Good	70.1(61)	87	48.3(42)	87	34.5(30)	87	31.0(27)	87
**HMIS, Supply Chain, and Guidelines**		(16.62)*		(5.18)		(4.22)		(2.74)
Poor	48.4(31)	64	35.9(23)	64	28.1(18)	64	25.0(16)	64
Fair	62.1(36)	58	46.6(27)	58	32.8(19)	58	31.0(18)	58
Good	80.5(66)	82	54.9(45)	82	43.9(36)	82	37.8(31)	82
**HCWs/1,000 Catch. Popn. (WHO Std.)**		(3.21)		(0.39)		(0.76)		(1.03)
<2.3	62.6(107)	171	45.6(78)	171	34.5(59)	171	30.4(52)	171
≥2.3	78.8(26)	33	51.5(17)	33	42.4(14)	33	39.4(13)	33
**Met 50% MSP+**		(18.73)*		(15.77)*		(15.71)*		(13.92)*
No	56.9(87)	153	38.6(59)	153	28.1(43)	153	24.8(38)	153
Yes	90.2(46)	51	70.6(36)	51	58.8(30)	51	52.9(27)	51
**Any Staff Received aFOMT in ≤1 year**		(0.45)		(0.25)		(0.04)		(0.79)
0-1	67.3(74)	110	48.2(53)	110	36.4(40)	110	34.5(38)	110
2+	62.8(59)	94	44.7(42)	94	35.1(33)	94	28.7(27)	94
**Infrastructure and Utility**		(6.03)*		(4.04)		(8.04)*		(5.42)
Poor	55.4(41)	74	39.2(29)	74	24.3(18)	74	23.0(17)	74
Fair	66.1(41)	62	45.2(28)	62	37.1(23)	62	32.3(20)	62
Good	75.0(51)	68	55.9(38)	68	47.1(32)	68	41.2(28)	68
**Tracer Drugs**		(66.30)*		(33.50)*		(32.4)*		(22.50)*
Poor	35.2(32)	91	24.2(22)	91	15.4(14)	91	15.4(14)	91
Fair	84.6(44)	52	61.5(32)	52	44.2(23)	52	38.5(20)	52
Good	93.4(57)	61	67.2(41)	61	59.0(36)	61	50.8(31)	61
**Tracer Equipment**		(64.08)*		(36.53)*		(31.70)*		(28.74)*
Poor	27.0(17)	63	15.9(10)	63	7.9(5)	63	6.3(4)	63
Fair	72.9(51)	70	54.3(38)	70	44.3(31)	70	38.6(27)	70
Good	91.5(65)	71	66.2(47)	71	52.1(37)	71	47.9(34)	71

NoHF: Number of Health Facilities; aFOMT: Financial and Operational Management Training; *p < 0.05; + Overall MSP standard for health workers.

Meeting at least 50% MSP human resources for health, availability of tracer drugs, and tracer equipment were significantly associated with 80% service provision across the three Tiers.

The higher the level of readiness regarding availability of tracer drugs and tracer equipment, the higher the proportion of health facilities that provide Tier 1, Tier 2, and Tier 3 services (p<0.05). The gender of the facility manager was significantly associated with meeting at least 80% service provision in all three Tiers.

Table 7 showed the adjusted multivariable logistic regression model outputs in each of the three Tiers of services and the combined Tiers. The data showed that the identified predictors of providing at least 80% Tier 1 services are facility management and financing, HMIS, supply chain, and guidelines; tracer drugs; and tracer equipment. The likelihood of providing at least 80% Tier 1 services was higher in facilities with fair level of facility management and financing readiness (aOR=4.78, 95% CI=1.18-19.23, p<0.05), good level of HMIS, supply chain, and guidelines readiness (aOR=3.13, 95% CI=1.17-8.33, p<0.05), compared to those in the poor category. Being in the fair (aOR=2.39, 95% CI=1.53-8.51, p<0.05) and good (aOR=4.47, 95% CI=1.94-14.74, p<0.05) level of readiness concerning the availability of Tracer Drugs promotes meeting a minimum of 80% Tier 1 service provision by the health facilities. A similar pattern shown by Tracer Drugs was observed in the pattern of relationship between the availability of Tracer Equipment and meeting a minimum of 80% Tier 1 service provision.

**Table 7:** Multivariable logistic regression model of the predictors of Tiers of Services’ provision by health facilities according to background characteristics.

Background	≥80% in Tier 1		≥80% in Tier 2		≥80% in Tier 3		≥80% in all Tiers	
Characteristics	uOR (95% CI)	aOR (95% CI)	uOR (95% CI)	aOR (95% CI)	uOR (95% CI)	aOR (95% CI)	uOR (95% CI)	aOR (95% CI)
**Gender of Officer in Charge**								
Female	1.00	1.00	1.00		1.00	1.00	1.00	1.00
Male	0.59 (0.32–1.05)^	0.82 (0.33–1.98)	0.77 (0.44–1.33)		0.55 (0.31–0.98)*	0.77 (0.38–1.54)	0.54 (0.29–0.98)*	0.74 (0.36–1.48)
**Facility Management and Financing**								
Poor	1.00	1.00	1.00	1.00	1.00		1.00	
Fair	2.87 (0.98–8.34)^	4.78 (1.18–19.23)*	3.03 (1.17–7.79)*	3.86 (1.26–11.80)*	1.61 (0.64–4.01)		2.06 (0.82–5.19)	
Good	1.77 (0.95–3.28)^	1.64 (0.6–4.01)	1.41 (0.78–2.55)	1.11 (0.53–2.27)	1.01 (0.54–1.86)		1.10 (0.58–2.08)	
**HMIS, Supply Chain, and Guidelines**								
Poor	1.00	1.00	1.00	1.00	1.00	1.00	1.00	
Fair	1.74 (0.84–3.59)	1.23 (0.43–3.48)	1.55 (0.75–3.21)	1.12 (0.46–2.71)	1.25 (0.58–2.69)	0.71 (0.28–1.82)	1.35 (0.61–2.99)	
Good	4.39 (2.10–9.15)*	3.13 (1.17–8.33)*	2.17 (1.10–4.24)*	1.18 (0.53–2.61)	2.00 (0.99–4.02)^	1.08 (0.47–2.45)	1.82 (0.88–3.75)	
**HCWs/1,000 Catch. Popn. (WHO Std.)**								
<2.3	1.00	1.00	1.00		1.00		1.00	
≥2.3	2.22 (0.91–5.41)^	1.80 (0.51–6.31)	1.27 (0.60–2.67)		1.40 (0.66–2.99)		1.49 (0.68–3.21)	
**Met 50% MSP**								
No	1.00	1.00	1.00	1.00	1.00	1.00	1.00	1.00
Yes	6.98 (2.62–18.54)*	0.44 (0.07–2.59)	3.82 (1.92–7.58)*	1.02 (0.28–3.62)	3.65 (1.88–7.07)*	1.27 (0.35–4.51)	3.41 (1.75–6.59)*	1.22 (0.34–4.28)
**Any Staff Received aFOMT in ≤1 yr**								
0-1	1.00		1.00		1.00		1.00	
2+	0.82 (0.46–1.46)		0.87 (0.50–1.51)		0.95 (0.53–1.68)		0.76 (0.42–1.38)	
**Infrastructure and Utility**								
Poor	1.00	1.00	1.00	1.00	1.00	1.00	1.00	1.00
Fair	1.57 (0.78–3.16)	0.93 (0.33–2.58)	1.28 (0.64–2.53)	0.74 (0.32–1.69)	1.84 (0.87–3.85)	1.32 (0.55–3.09)	1.60 (0.74–3.41)	1.07 (0.45–2.52)
Good	2.42 (1.18–4.94)*	1.26 (0.46–3.45)	1.97 (1.01–3.84)*	1.17 (0.52–2.61)	2.77 (1.35–5.64)*	1.96 (0.87–4.39)	2.35 (1.13–4.85)*	1.62 (0.72–3.62)
**Tracer Drugs**								
Poor	1.00	1.00	1.00	1.00	1.00	1.00	1.00	1.00
Fair	4.14 (1.25–6.15)*	2.39 (1.53–8.51)*	5.02 (2.40–10.48)*	3.98 (1.55–10.20)*	4.36 (1.98–9.61)*	1.89 (0.76–4.69)	3.44 (1.54–7.63)*	1.45 (0.57–3.64)
Good	5.27 (1.73–15.03)	4.47 (1.94–14.74)*	6.43 (3.13–13.19)*	4.89 (1.75–13.59)*	7.92 (3.68–17.01)*	2.89 (1.10–7.54)*	5.68 (2.66–12.14)*	1.79 (0.67–4.69)
**Tracer Equipment**								
Poor	1.00	1.00	1.00	1.00	1.00	1.00	1.00	1.00
Fair	3.56 (1.37–15.63)*	3.49 (1.34–9.05)*	6.29 (2.76–14.34)*	3.57 (1.07–6.19)*	3.22 (1.29–8.78)*	5.17 (1.68–15.83)*	3.26 (1.01–8.42)*	2.38 (1.92–11.14)*
Good	4.83 (2.14–16.01)*	4.11 (2.45–15.79)*	4.15 (1.8–9.58)*	3.78 (0.96–6.40)	3.62 (1.51–6.20)*	4.74 (1.44–15.56)*	4.55 (1.44–9.31)*	3.93 (1.96–8.49)*
**Met MSP Standard**	1.63 (1.37–1.94)*	1.29 (0.95–1.73)	1.36 (1.19–1.55)*	1.13 (0.88–1.44)	1.34 (1.17–1.53)*	1.07 (0.82–1.38)	1.32 (1.15–1.50)*	1.07 (0.82–1.38)

aFOMT: At least two Financial and Operational Management Training; *p < 0.05; ^p < 0.10.

The data further revealed that the identified predictors of providing at least 80% Tier 2 services are availability of facility management and financing, tracer drugs, and tracer equipment, while those of Tier 3 services are only availability of tracer drugs and tracer equipment. The common predictors across the service Tiers were the availability of Tracer drugs and Tracer equipment. However, the only identified factor influencing provision of at least 80% Tier 1, Tier 2, and Tier 3 (combined) services was tracer equipment and the odds was higher in health facilities with fair (aOR=2.38, 95% CI=1.92-11.14, p<0.05) and good (aOR=3.93, 95% CI=1.96-8.49, p<0.05) level of readiness than those with poor.

## Discussion

The numerous challenges with access and quality of care, including insufficient skilled personnel, poor management and finance, scarcity of HMIS tools and guidelines, dilapidated infrastructure and utilities, and lack of tracer equipment and tracer drugs in Nigeria have been established by earlier studies [12,15,17,25–27]. However, explicit information is required to inform policy actions that will alleviate such challenges. Therefore, the current study examined the relationship between facility readiness and health services’ provision in the prioritized Nigerian PHC facilities. The PHC ward development and facility management committees in Nigeria hold health care workers accountable for their performance, including monitoring absenteeism, quality of services, and expenditures [28,29]. The committees monitor the quality of care, address genuine concerns of the staff and clients, drive resource mobilization, ensure active community participation, and vocalize the community’s concerns [28,30]. In the current study, almost all the PHCs investigated have ward development and facility management committees. This finding corroborates the outcome of national and sub-national studies previously conducted in Nigeria [30–33]. Despite the striking availability of ward development and facility management committees in almost all the studied facilities, the persistent poor performance of PHC in Nigeria [15,26,33] should be of great concern. This situation could be attributed to issues, including, but not limited to, transparency, corruption, participatory selection, training and capacity building, representation and inclusion, motivation, and clear declaration of roles among the ward development and facility management committees [34,35].

The World Health Organization (WHO) emphasized the need to develop and strengthen HMIS to improve health care delivery. The effective use of HMIS often leads to the transformation of data into evidence-based decisions, effective management of resources, acceleration of operational efficiency, reduction of clinical errors, promotion of active surveillance, and monitoring progress against global health targets [36]. Consequently, the organization provides guidance, tools, strategic policy frameworks, and builds partnerships to assist countries in developing and strengthening their health systems [36–38]. In the current study, a high proportion of the assessed PHC facilities have HMIS tools/registers, quarterly financial reports for BHCPF, standard operating procedures (SOPS), integrated management of childhood illnesses guidelines, and immunization schedule guidelines. However, the facilities exhibited poor accounting practices in developing monthly financial reports for SCHMA/SHIA funds, updated BHCPF annual quality improvement plan, and expenditures aligned with the business plan. Summarily, a low proportion of the health facilities exhibited a good level of readiness relative to management and finance, and HMIS tools/registers, supply chain tools/registers, and guidelines. These findings are consistent with the previous studies conducted in Nigeria and elsewhere in sub-Saharan Africa [14,17,39,40]. The poor accounting practices exhibited by some of the health facilities could be due to a lack of training, as demonstrated by low exposure to financial or operational management training by the health facilities’ staff. Other possible reasons that were not established in this study, but are peculiar to Nigeria, are intentional fraud/unethical conduct, pressure to meet unrealistic financial goals, unmotivated staff, poor internal controls, and failure to stay updated with regulatory changes [41,42]. The availability of HMIS tools/registers and guidelines in a large proportion of the health facilities is creditable, and this is an indication of compliance with the NPHCDA policy and other declarations which emphasized strengthening the HMIS to improve data availability and utilization for evidence-based decision-making at all levels of the health system by the PHC facilities [36,43].

Achieving quality health care services, delivering effective interventions, and improving health outcomes requires a skilled and adequate health workforce [44]. However, low-income countries such as Nigeria face challenges in training, deploying, and retaining their skilled health workers [34,42,45]. The WHO recommends a minimum threshold of 2.3 skilled health workers per 1,000 population for a country to achieve adequate coverage for essential health services [46]. This was part of the WHO global strategy on human resources for health agenda, chiefly to ensure a workforce that is fit for purpose to attain the targets of the SDGs. Despite this recommendation about a decade ago, only six out of ten health facilities investigated by this study met the minimum skilled health care workers density (WHO standard (2.3 HCWs/1000 population)) to achieve 80% skilled birth attendant coverage, while none of the health facilities met the MSP standard for health care workers in all eleven cadres of health care workers, which are consistent with the available findings [44,46,47]. The finding may be related to the brain drain occurrence in the health sector, primarily driven by low wages, insecurity, poor working conditions, and limited opportunities for career advancement [2,47,48]. Many developed countries offer a more secure and predictable environment for health care workers [49–51]. Additionally, the few available skilled health care workers in Nigeria preferred to work in the secondary and tertiary health facilities in the major urban areas, thus pulling them from the PHC facilities.

Environmental health care facilities’ amenities, such as handwashing with soap and running water, and functioning toilets, are available in a larger proportion of the investigated health facilities. However, it is worrisome that only one-tenth of the facilities have at least a functional and dedicated ambulance, and access to any vehicle for emergency transport services was not available in many health facilities. The health facilities are limited in tracer equipment, including a sphygmomanometer, pulse oximeter, thermometer, sterilizers, angle poise lamp, ambu bag mask, and nebulizer, particularly in the specific units. Generally, a low proportion of the health facilities exhibited a good level of readiness relative to infrastructure and utility, tracer equipment, and tracer drugs as reported by similar studies [5,52–54]. The low level achieved could be attributed to corruption, diversion of resources for health, misplacement of health priorities, and mismanagement of funds meant for the procurement of tracer drugs and equipment [2,55,56]. Passive monitoring of the implementation of the ongoing reforms, such as the revitalization of 17,000 PHCs through the World Bank IMPACT project, BHCPF, and other funding sources, is another probable reason for the low PHC readiness found in this study. Poor access to ambulances and emergency transport services at the studied PHC facilities mirrors the prevailing Nigerian health facilities situation [30,33]. A restructuring approach may be needed to address the contemporary gaps in the Nigerian PHCs. This calls for swift action by the appropriate government authorities and agencies.

Primary health care facilities are the first point of contact within the Nigerian health system, and the main services they provide include: maternal and child health, adolescent health, men’s health, care of the elderly, minor life-saving services, oral health, and mental health [46,57]. Others include voluntary counselling and screening, family planning, immunization, drug dispensing, environmental health services and food control, nutrition education and micronutrient supplementation, registration of births and deaths, health education, referral system, and community development activities [58,59]. These services are classified into Tier 1, Tier 2, and Tier 3 based on their operational levels as presented in the method section. There have been proclamations that each PHC provide at least 80% Tier 1, Tier 2, and Tier 3 services to achieve the universal health coverage agenda (SDG 3.8 target) by 2030 [6]. Our study revealed that a higher proportion of the PHC facilities provided at least 80% Tier 1 service compared to Tier 2 or Tier 3 services, while a few met the provision of at least 80% of services in each of the three service Tiers. The provision of at least 80% Tier 2 and Tier 3 services occurred in less than half of the health facilities. These findings corroborate the documented information about the state of service provision in the Nigerian PHC facilities [16,31,55]. The probable reasons for poor health service delivery in Nigeria are multifaceted, often linked with underfunding, understaffing, poor infrastructure, inadequate skilled staff, weak leadership, and some underlying sociocultural beliefs and practices.

Drugs and equipment availability are part of the global health development goals [8,60,61]. They are critical to effective treatment, disease management, health care costs, patient outcomes, and health system efficiency. Deficiencies in access to essential medications and equipment at the PHC facilities can promote treatment delays, disease progression, treatment failure, increased hospitalization, increased risk of adverse drug reactions, reduced patient satisfaction, and increased mortality rates [62,63]. In this study, the common factors influencing the provision of at least 80% of services across the three Tiers were the readiness concerning tracer drugs and equipment. Achieving at least a fair level of readiness regarding tracer drugs and equipment was protective for delivering at least 80% of any of the three service Tiers. These findings align with the established literature in Bangladesh, Nigeria, and elsewhere [16,64,65]. The availability of drugs and equipment is essential for efficient health care service delivery. If the health personnel were available and exposed to regular training but without the availability of essential drugs and equipment, service provision would be stagnated; hence, access to services could be compromised [66,67]. Therefore, addressing drug and equipment availability-related challenges like supply chains, funding, and the implementation of effective inventory management practices may improve health care service delivery and build a more resilient health care system in the studied health facilities. This can be achieved if the disbursed fund aimed at strengthening the provision of essential health services through the BHCPF is used wisely.

### Strengths and limitations of the study

This study has provided useful information regarding the state of PHC health facilities concerning readiness and provision of health services. Identifying these critical gaps is vital for designing interventions to enhance PHC performance in Nigeria. The findings can provide information that is helpful to the restructuring, provision of corrective measures, including adjustments of the monitoring and evaluation plan for the IMPACT Project and the BHCPF in Nigeria. This may accelerate the plan of the government, agencies, and their implementing partners to revamp the PHCs in Nigeria. Additionally, other PHC facilities in Nigeria can learn from the best practices documented in the study. However, the study was conducted only among PHC facilities in six prioritized states out of 36 states and the FCT in Nigeria; thus, a relatively underrepresented population whose results cannot be used for the entire country. The analysis might not permit a state-level analysis due to the low sample size allotted to each state. The causality between facility readiness and service provision cannot be established due to the cross-sectional design approach used in this study. Therefore, the prospective readers of this article should interpret the findings with caution.

### Implications

These findings have direct implications for PHC policy and programming in Nigeria. State primary healthcare development agencies and the National Primary Health Care Development Agency (NPHCDA) should prioritize the consistent availability of tracer drugs and functional tracer equipment, as these were the strongest and most consistent predictors of service provision across all three tiers. BHCPF and other capitation-based financing mechanisms should be reviewed to ensure that quarterly disbursements are sufficient and timely for facilities to replenish essential commodities and maintain equipment. Given the persistent shortfall in the health workforce relative to WHO and Minimum Service Package standards, state human-resource-for-health plans should explore task-shifting, task-sharing, and targeted recruitment or deployment incentives, particularly for underserved wards. Finally, because facility readiness across the six domains was strongly associated with service provision, PHC supervision and quality-improvement systems should track readiness indicators routinely, rather than relying on periodic facility assessments alone, to allow for timely corrective action.

## Conclusion

The readiness level of the PHC facilities with respect to the five domains, viz. management and finance, HMIS tools/registers, supply chain tools/registers and guidelines, infrastructure and utility, tracer equipment, and tracer drugs, can be considered as poor. Good PHC facility readiness augments the provision of at least 80% Tier 1, Tier 2, and Tier 3 services. However, the availability of tracer drugs and tracer equipment is the common predictor of providing at least 80% of services in all three service Tiers in the study area. The findings highlight important gaps in service delivery and access to health services at the PHC facilities. Weaknesses and gaps in essential drugs and equipment were common in the health facilities. Therefore, adequate provision of tracer drugs and equipment will improve service delivery in the Nigerian PHC facilities. To achieve health equity and universal health coverage in Nigeria, the stakeholders should prioritize reforms in financing and procurement to strengthen PHC readiness.

## Supporting information

S1 Table The three tiers of services provided at primary health care (PHC) level in Nigeria. (DOCX)

S2 Table Primary health care facilities service readiness domains and their components. (DOCX)

S1 File Dataset. (XLSX)
