# Supplementary material for: Assessing primary health care facilities in six prioritized Nigerian states: Linking facility readiness with service availability
Source: PLoS One. 2026 Oct 13;21(10):e0360425. doi: 10.1371/journal.pone.0360425 (PMC13660913; doi:10.1371/journal.pone.0360425)
Supplement: Supplementary file 1 [file pone.0360425.s001.docx]

**Table 1. The Three Tiers of Services Provided at Primary Health Care (PHC) Level in Nigeria**

| **Tier** | **Subcategory** | **Services** |
| --- | --- | --- |
| **Tier 1** | Maternal health | • Antenatal care check-up  • Antenatal care laboratory test, HTC  • Provide folate, 2 doses of IPT to pregnant women  • Provide pre-referral treatment for severe anaemia  • PMTCT/HIV – no-cost ARV  • Provide treatment for syphilis  • Treat other STDs apart from syphilis  • Basic obstetric care (normal delivery)  • Provide Vitamin A to post-partum women  • Provide pre-referral treatment for neonatal tetanus  • Refer complicated labour/delivery cases |
|  | Newborn care | • Newborn care  • Provide pre-referral treatment for neonatal tetanus  • Prevent newborn infection  • Prevent and manage newborn hypo/hyperthermia  • Early asphyxia identification and management  • Prevent and manage ophthalmia neonatorum  • Identification and management of sick newborns |
|  | Child health | • Management of childhood illness (IMCI)  • Monitor ORT/feeding for diarrhoea  • Treat acute respiratory infection with antibiotics |
|  | Family planning | • Counselling and motivation for family planning  • Dispensing of male and female condoms  • Dispensing of contraceptives  • Dispensing injectable  • Insertion of IUCD |
|  | Immunization | • Identification of eligible pregnant women and children for immunization  • Provision of routine immunization, TT, BCG, OPV, DPT, YF, MV, etc.  • Participation in immunization campaigns  • Immunization trends follow-up  • Assist in the management of Adverse Events Following Immunization (AEFI)  • Assist in the identification of Acute Flaccid Paralysis (AFP) |
|  | Malaria | • Antimalarial drugs for fever in malaria  • Malaria prevention – provide ITNs  • IPT for pregnant women |
|  | Nutrition | • Provision of micronutrients, Vitamin A supplementation for children  • Deworming for all ages  • Screening for nutrition-related problems (PEM, anaemia, goiter)  • Nutritional education, including food hygiene  • Nutrition assessment |
| **Tier 1** | Community health | • Community mobilization for health |
| **Tier 2** | Newborn care | • Care of preterm and/or low birth weight newborn |
| **Tier 2** | HIV/AIDS and sexually transmitted infections | • Management of opportunistic infections  • Syndromic management of STIs |
| **Tier 2** | Tuberculosis | • Confirm the diagnosis for TB through laboratory tests  • Provide DOTs to cases |
| **Tier 2** | Cardiovascular health | • Screening and diagnosis of hypertension  • Prevention of adverse cardiovascular disease (stroke, heart attack, rheumatic heart disease)  • Prevention of sore throat, fever, and joint pains to rule out acute rheumatic fever  • Counselling on lifestyle management based on findings from the risk assessment  • Support for self-management of cardiovascular diseases |
| **Tier 2** | Adolescent and adult immunization | • Life course vaccine for adolescents and adults (HBV/HPV) |
| **Tier 2** | Referral | • Referral to secondary/tertiary health facilities for further management |
| **Tier 3** | Emergency obstetric care | • Emergency obstetric care: post-abortion care  • Manage Post Partum Haemorrhage  • Manage Puerperal Sepsis |
| **Tier 3** | Oral health | • Advice on the care of the mouth and teeth |
| **Tier 3** | Diabetes | • Screening for diabetes  • Provide treatment for diabetes cases |
| **Tier 3** | Chronic respiratory disease | • Chronic Respiratory Disease Control  • Management of asthmatic attack, asthma, and Chronic Obstructive Pulmonary Disease |
| **Tier 3** | Mental health | • Mental Health Management |

*Note: The source table does not explicitly name the subcategories. The subcategories in this structured version were assigned from the service groupings shown in the source table; the service wording has been retained as closely as possible.*
