# Supplementary material for: Assessing primary health care facilities in six prioritized Nigerian states: Linking facility readiness with service availability
Source: PLoS One. 2026 Oct 13;21(10):e0360425. doi: 10.1371/journal.pone.0360425 (PMC13660913; doi:10.1371/journal.pone.0360425)
Supplement: Supplementary file 2 [file pone.0360425.s002.docx]

**Table 2. Primary Health care Facilities service readiness domains and their components**

| **S/N** | **Domains** | **Components (Sub-domains)** | **NoV** |
| --- | --- | --- | --- |
| 1. | Health Facility Management and | Organization and Management Characteristics | 6 |
|  | Financing | Documentation and Organizational Resources | 5 |
|  |  | Budgeting & Expenditure Tracking | 3 |
|  |  | Financial Documentation | 1 |
|  |  | Accounting System Practices | 4 |
|  |  | Total | 19 |
| 2. | HMIS Tools/Registers, Supply | HMIS tools/registers | 7 |
|  | Chain Tools/Registers, and | Supply Chain Management Tools | 8 |
|  | Guidelines | Guidelines | 6 |
|  |  | Total | 21 |
| 3. | Human Resources for Health | MSP standard for Health care Workers* | 1 |
|  |  | Skilled Health Care Workers (HCWs) density** | 1 |
|  |  | Total | 2 |
| 4. | Infrastructure and Utility | Power Source | 4 |
|  |  | Water Source | 4 |
|  |  | Physical Infrastructure | 10 |
|  |  | Transportation and communication | 2 |
|  |  | Total | 20 |
| 5. | Availability of Tracer Equipment | Outpatient Department (OPD) | 8 |
|  |  | Minor Theater/Causality | 9 |
|  |  | Immunization | 6 |
|  |  | Antenatal Care | 8 |
|  |  | Total | 31 |
| 6. | Availability of Tracer Drugs | Total | 15 |

*NoV: Number of variables; HMIS: Health Management Information System; MSP: Minimum Service Package; *at least 1 Medical Doctor, at least 1 CHO, at least 4 Nurses/Midwives, at least 3 CHEWs, and at least 6 JCHEWs. at least 1 EHO, at least 2 Pharmacy Technicians, at least 2 Laboratory Technicians, and at least 2 HA, at least 2 MRO, and at least 2 security staff; **WHO standard 2.3 HCWs/1000 population*
